# Current density imaging sequence for monitoring current distribution during delivery of electric pulses in irreversible electroporation

**DOI:** 10.1186/1475-925X-14-S3-S6

**Published:** 2015-08-27

**Authors:** Igor Serša, Matej Kranjc, Damijan Miklavčič

**Affiliations:** 1 Institut "Jozef Stefan", Jamova cesta 39, SI-1000, Ljubljana, Slovenia; 2 University of Ljubljana, Faculty of electrical engineering, Tržaška 25, SI-1000, Ljubljana, Slovenia

**Keywords:** Irreversible electroporation, tissue conductivity, magnetic resonance imaging, current density imaging, magnetic resonance electrical impedance tomography

## Abstract

**Background:**

Electroporation is gaining its importance in everyday clinical practice of cancer treatment. For its success it is extremely important that coverage of the target tissue, i.e. treated tumor, with electric field is within the specified range. Therefore, an efficient tool for the electric field monitoring in the tumor during delivery of electroporation pulses is needed. The electric field can be reconstructed by the magnetic resonance electric impedance tomography method from current density distribution data. In this study, the use of current density imaging with MRI for monitoring current density distribution during delivery of irreversible electroporation pulses was demonstrated.

**Methods:**

Using a modified single-shot RARE sequence, where four 3000 V and 100 μs long pulses were included at the start, current distribution between a pair of electrodes inserted in a liver tissue sample was imaged. Two repetitions of the sequence with phases of refocusing radiofrequency pulses 90° apart were needed to acquire one current density image. For each sample in total 45 current density images were acquired to follow a standard protocol for irreversible electroporation where 90 electric pulses are delivered at 1 Hz.

**Results:**

Acquired current density images showed that the current density in the middle of the sample increased from first to last electric pulses by 60%, i.e. from 8 kA/m^2 ^to 13 kA/m^2 ^and that direction of the current path did not change with repeated electric pulses significantly.

**Conclusions:**

The presented single-shot RARE-based current density imaging sequence was used successfully to image current distribution during delivery of short high-voltage electric pulses. The method has a potential to enable monitoring of tumor coverage by electric field during irreversible electroporation tissue ablation.

## Background

Clinical applications of electroporation such as electrochemotherapy (ECT) [1-3] and irreversible electroporation (IRE) tissue ablation [4-6] are emerging procedures in solid tumor treatment [7]. Both procedures are based on applying short high-intensity electric field pulses to cells, whose membrane in response becomes either temporarily permeable to chemotherapeutic drugs (ECT) or gets destroyed (irreversible tissue ablation) [8]. Since accurate coverage of the treated tissue with sufficiently large electric field presents one of the most important conditions for successful electroporation [9,10], a method for reconstruction of electric field distribution based on magnetic resonance electrical impedance tomography (MREIT) was suggested [11]. MREIT is a magnetic resonance imaging technique that is primarily used for reconstruction of electrical conductivity of imaging samples [12]. Feasibility of this method to determine electric field distribution during electroporation was demonstrated in an agar phantom [11], in liver tissue *ex vivo *[13], *in silico *[14] and recently during electroporation of mouse tumor *in vivo *[15].

MREIT is enabled by Current Density Imaging (CDI), an MRI modality designed to detect electric currents via temporal change of magnetic field that is induced by the currents [16]. In CDI, the magnetic field change is recorded in a phase of a MR image, which is then used to calculate current density using Ampere's law [17]. Since its introduction in 1989 CDI has become a versatile method for study electrical conductivity properties at different frequency regimes of the applied currents: direct currents [16], alternating currents [18] and radio-frequency currents [19]. The method was successfully applied to different biological samples [20,21] as well as materials [22]. Recent developments in CDI combined with MREIT algorithms for imaging tissue conductivity tensor [23,24]. CDI and MREIT algorithms have also already been used as a monitoring tool and for guidance in treatment by RF ablation [25]. Due to low conductivity of biological samples and usual need for sample rotation in the magnet to obtain all needed components of current induced magnetic field change, CDI is difficult to perform *in vivo *and is limited to only a few applications and sample/electrode geometries. One such application, which is also a topic of this study, is the use of CDI to monitor current density distribution in the sample during delivery of electric pulses in IRE. In previous studies [11,13-15] we employed CDI to obtain current density distribution during electroporation for reconstruction of electric field distribution by means of MREIT algorithms using electric pulses with parameters that are applied in reversible electroporation applications such as ECT; i.e. pulse repetition rate of 5 kHz and amplitudes between 1000 V and 1500 V. IRE applications such as irreversible tissue ablation would also benefit from the method that would enable monitoring of the electroporation process through the reconstruction of electric field distribution *in situ *using MREIT. ECT and IRE are not really different in voltage and current amplitude but in pulse repetition frequency and most of all in number of pulses delivered. Since MREIT algorithms depend on the quality of the measured current density distribution, a new CDI sequence, optimized for currents delivered by electroporation pulses used in IRE, is needed. Therefore, the aim of this study was to develop a new CDI sequence that would enable monitoring of current density distribution during application of electric pulses with parameters that are commonly used in IRE tissue ablation procedures, i.e. repetition rate of approximately 1 Hz and an amplitude of 3000 V [26,27].

## Methods

### Current density imaging sensitivity

**Figure 1:**
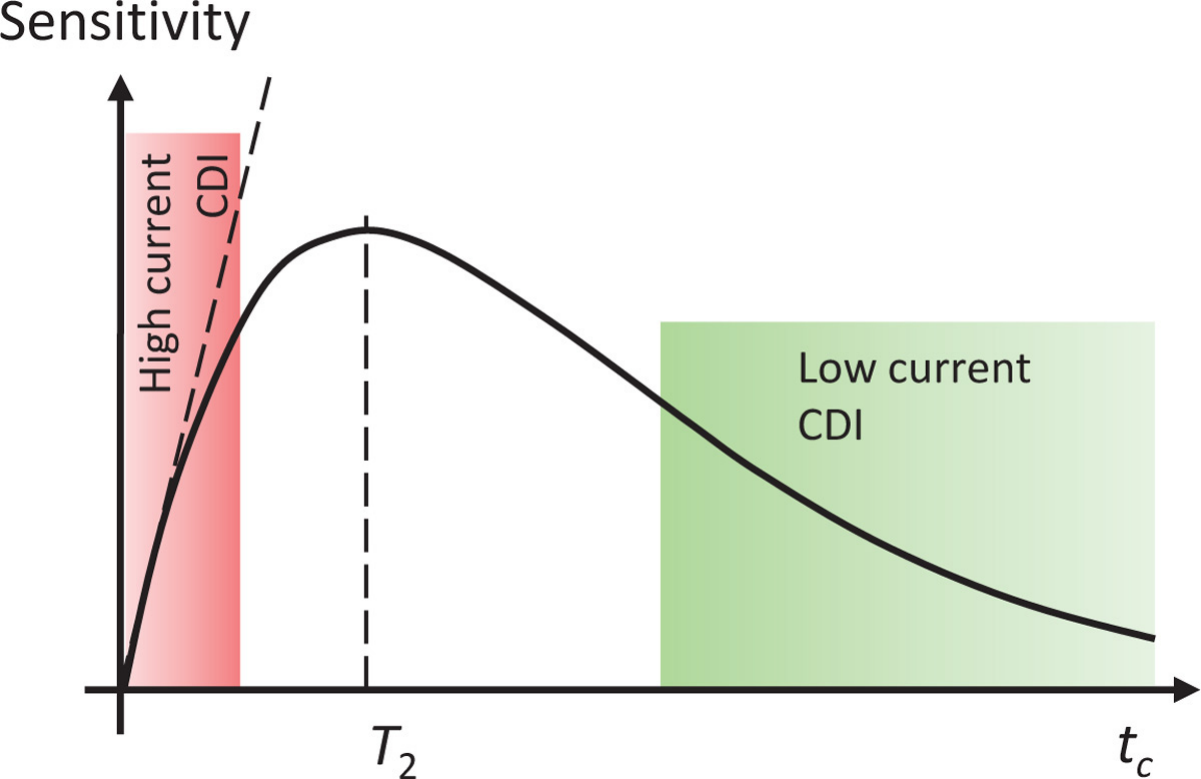
**Sensitivity of a CDI sequence is proportional to the duration of applied electric pulses *t_c _*multiplied by *SNR *of the conventional (magnitude) MR image**. As the latter is in most sequences exponentially decaying with the relaxation time *T*_2 _the highest sensitivity is obtained at *t_c _*= *T*_2_. The condition is in practice difficult to meet as most biomedical CDI applications require either very short and high-current pulses (electroporation, IRE) or very long and low-current pulses (CDI of physiological currents).

It has been shown [17,28] that the sensitivity of current density imaging (CDI) is proportional to the total duration of applied electric pulses *t_c _*in the sequence and to the signal-to-noise ratio (*SNR*) of the magnitude image obtained by the sequence. The relation follows from the phase-based detection of CDI. Namely, in CDI current density is calculated using Ampere's law from a known magnetic field change due to applied electric current pulses. The magnetic field change is measured from the image phase shift that is proportional to *t_c_*, while the error of the measured magnetic field change (standard deviation of the measured phase shift) is inversely proportional to *SNR*. Therefore, CDI sensitivity is proportional to *t_c _· SNR*. In most cases, i.e. for all spin-echo based imaging sequences, image signal decays exponentially with the time between signal excitation and its acquisition. If it is also assumed that this time is approximately equal to *t_c _*then it follows that *SNR *is proportional to exp(-*t_c_*/*T*_2_) and CDI sensitivity to *t_c _*exp(-*t_c_*/*T*_2_); here *T*_2 _is the spin-spin relaxation time of the sample. As presented in Figure 1, CDI sensitivity has its peak at *t_c _*= *T*_2_, which is also most favorable condition for performance of CDI experiments. In the case electroporation monitoring by CDI, the electric pulses are so short that *T*_2 _relaxation factor in the CDI sensitivity formula is equal to unity so that the sensitivity is proportional only to the duration of applied electric pulses (dashed line in red area in Figure 1).

### CDI sequence for monitoring irreversible electroporation

**Figure 2:**
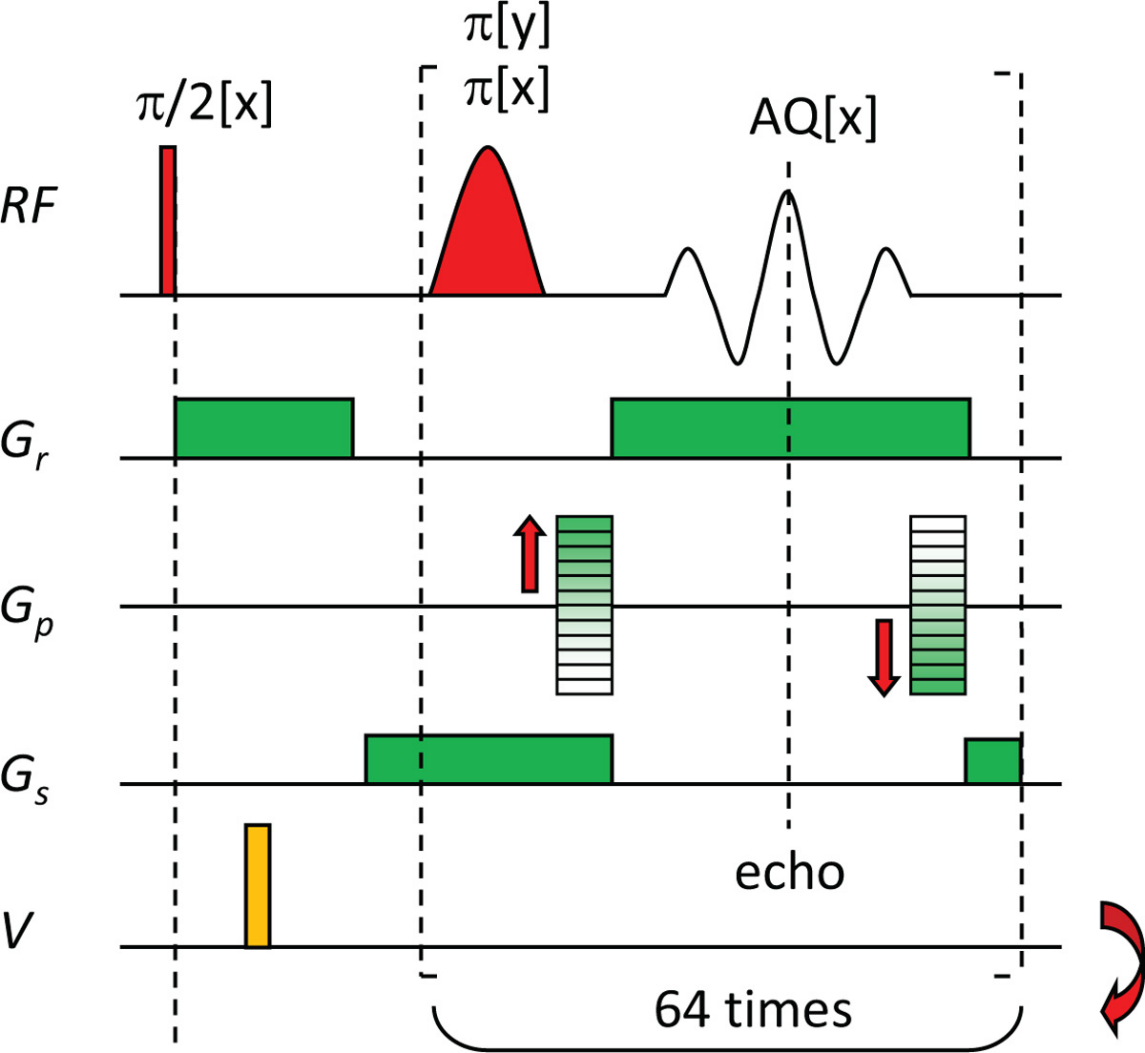
**Two-shot RARE pulse sequence that was used for monitoring IRE**. The sequence consists of a current encoding part with a short (100 μs long) high-voltage electroporation pulse (yellow) delivered immediately after the nonselective 90° radiofrequency (RF) excitation pulse. In the second part of the sequence signal acquisition is performed using the single-shot RARE signal acquisition scheme that includes standard execution of readout (Gr), phase-encoding (Gp) and slice-selection (Gs) magnetic field gradients. Due to auxiliary phase encoding induced by the electric pulse, the RARE sequence is repeated twice, each time with a different phase of the refocusing pulses (0°and 90°), and the corresponding signals are co-added.

Delivery of electroporation pulses for IRE was monitored by the CDI sequence based on the single-shot RARE image acquisition scheme [29] (Figure 2). The sequence had a single electric pulse inserted between the signal excitation π/2 RF pulse and the first shaped refocusing π RF pulse. The second part of the sequence is a multi-echo signal acquisition loop in which signal from all lines of the image k-scape was acquired. The signal from each echo was used to sample one k-space line. As the application of electric pulse resulted in an auxiliary phase shift that would destabilize the RARE signal acquisition scheme its stabilization was assured by a modification of the RARE signal acquisition described in [30]. Briefly, the stabilization is based on dual-repetition of the single-shot RARE image acquisition block, i.e. the two-shot RARE sequence. First time it is repeated with phases of the refocusing π RF pulses equal to the phase of the excitation π/2 RF pulse and in the second repetition the phases of the refocusing π RF pulses are shifted by 90° with respect to the phase of the excitation π/2 RF pulse. Finally, the acquired signals of both repetitions are co-added and the image is then reconstructed using a standard reconstruction procedure for RARE data sets.

**Figure 3:**
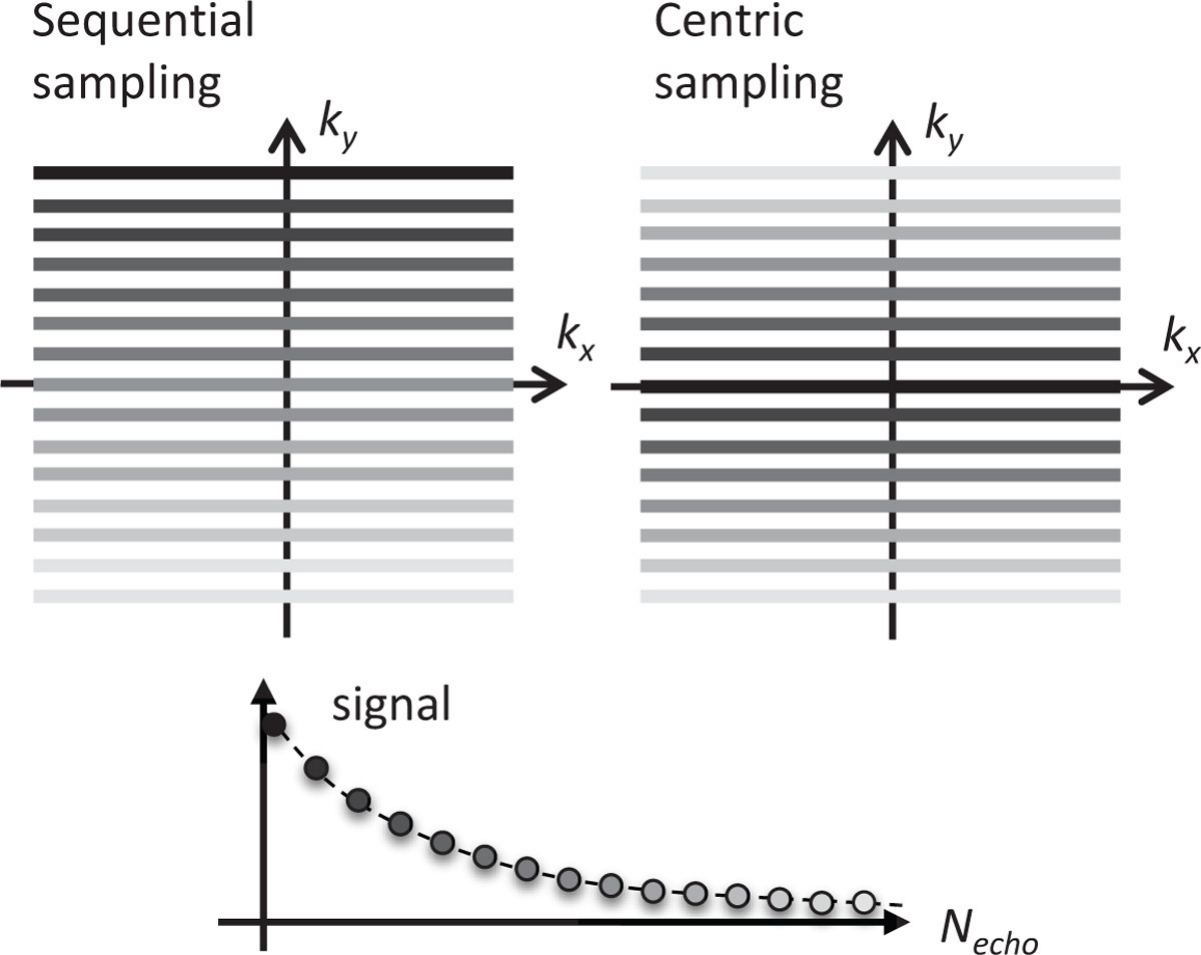
**RARE sequence enables different modes of signal acquisition in lines of the *k*-space, where k-space is the Fourier transform of the MR image measured**. Figure depicts two most often used modes where lines are ordered sequentially (left) or centric (right). The darkest line is sampled first and has the lowest signal reduction due to *T*_2 _signal relaxation. Lines sampled after the first have a progressive *T*_2 _signal relaxation effect that is depicted by an increasing brightness of lines (2D *k*-space) or dots (signal vs. *N_echo _*plot). The centric approach has an advantage over the sequential approach in a higher *SNR *due to the dominant signal in the *k*-space center. Therefore, the centric approach was chosen for CDI applications as it provides good CDI sensitivity.

The RARE sequence can be ran with different ordering of sampling signal from k-space lines. The lines can be ordered in sequential or centric order, as it is shown in Figure 3; however, other less frequently used orders are possible as well. The sequential order seems most obvious and the easiest to implement, but it is unfortunately not an optimal choice for CDI applications due to a need for high sensitivity rather than constrast. In this sampling order center of the k-space, which contains most image's low-resolution data and has the highest k-space signal, is sampled in the middle of the image acquisition loop. The signal from the central part is thus already suppressed due to *T*_2 _relaxation. Therefore, the phase noise and also CDI sensitivity is not as good as it would be if center of the k-space would be sampled first and would thus not be largely affected by the *T*_2 _relaxation. For this reason the centric sampling order is better than the sequential one. By using the centric sampling order the obtained image has better *SNR *and therefore also has better CDI sensitivity.

### Experimental

Proposed method of measuring current density distribution during IRE therapy was evaluated on *ex vivo *beef liver tissue which was primarily intended for human consumption. Liver tissue was obtained from a slaughterhouse which operates in accordance to Slovenian law. Experiments were in compliance with the slaughterhouse as all of their goods are produced strictly for human consumption. The process of slaughtering is regulated by Rules on animal protection and welfare at slaughter (Ur. l. RS, N. 5/2006) which ensures ethical standards of slaughtering procedure and is in compliance with European Union Council directive on the protection of animals at the time of slaughter or killing (93/119/EC). Temperature of the liver tissue was maintained at 4°C before the beginning of experiment when they were allowed to heat up to the room temperature, while time between slaughtering and the experiment was between 24 and 120 hours. Tissues were sectioned in cylindrical and flat shaped samples with a diameter of 21 mm and height of 4 mm in order to fit into an acrylic glass container designed for CDI experiments. Two self-built cylindrically shaped platinum-iridium electrodes with a diameter of 1 mm were inserted in the tissue. The distance between the electrodes was 14 mm. The sequence of 90 high voltage electric pulses with an amplitude of 3000 V and a duration of 100 μs at a repetition rate of 1 Hz were delivered between the electrodes. Electric pulses were delivered using customized Cliniporator Vitae (IGEA, Carpi, Italy) pulse generator. The electric pulse generator has 6 independently controlled and electrically insulated outputs each providing rectangular pulses with amplitudes up to 3000 V and 50 A maximum current. The generator is also capable of measuring the output voltage at 3 % precision. The current of electric pulses was measured with an oscilloscope (Wavesurfer 422, LeCroy, USA) using a current probe (AP015, LeCroy, USA) clamped around a wire connecting an electrode and the electric pulse generator. All experiments were repeated three times. The sample was replaced with a fresh one after each IRE pulse series delivery to ensure identical initial conditions in all electroporation experiments.

Two-shot RARE pulse sequence for monitoring IRE was performed on a 2.35 T horizontal bore small animal MRI scanner. The scanner was based on an Oxford superconducting magnet (Oxford Instruments, Abingdon, UK), an Apollo NMR/MRI spectrometer (Tecmag Inc., Houston TX, USA) and MRI probes for MR microscopy (Bruker, Ettlingen, Germany). The gradient hardware was able to deliver gradient pulses of up to 250 mT/m at the switching time of 200 μs. The spectrometer had several programmable TTL outputs that serve for a control of peripheral devices, one of these was used to trigger electroporation pulses on the electric pulse generator synchronously with the imaging sequence. Parameters of the sequence were the following: field of view 30 mm, imaging matrix 64 by 64, RARE factor 64, slice thickness 4 mm, echo time 2.64 ms, and repetition time 1 s. Centric sampling ordering of k-space lines was used to maximize CDI sensitivity.

## Results and discussion

**Figure 4:**
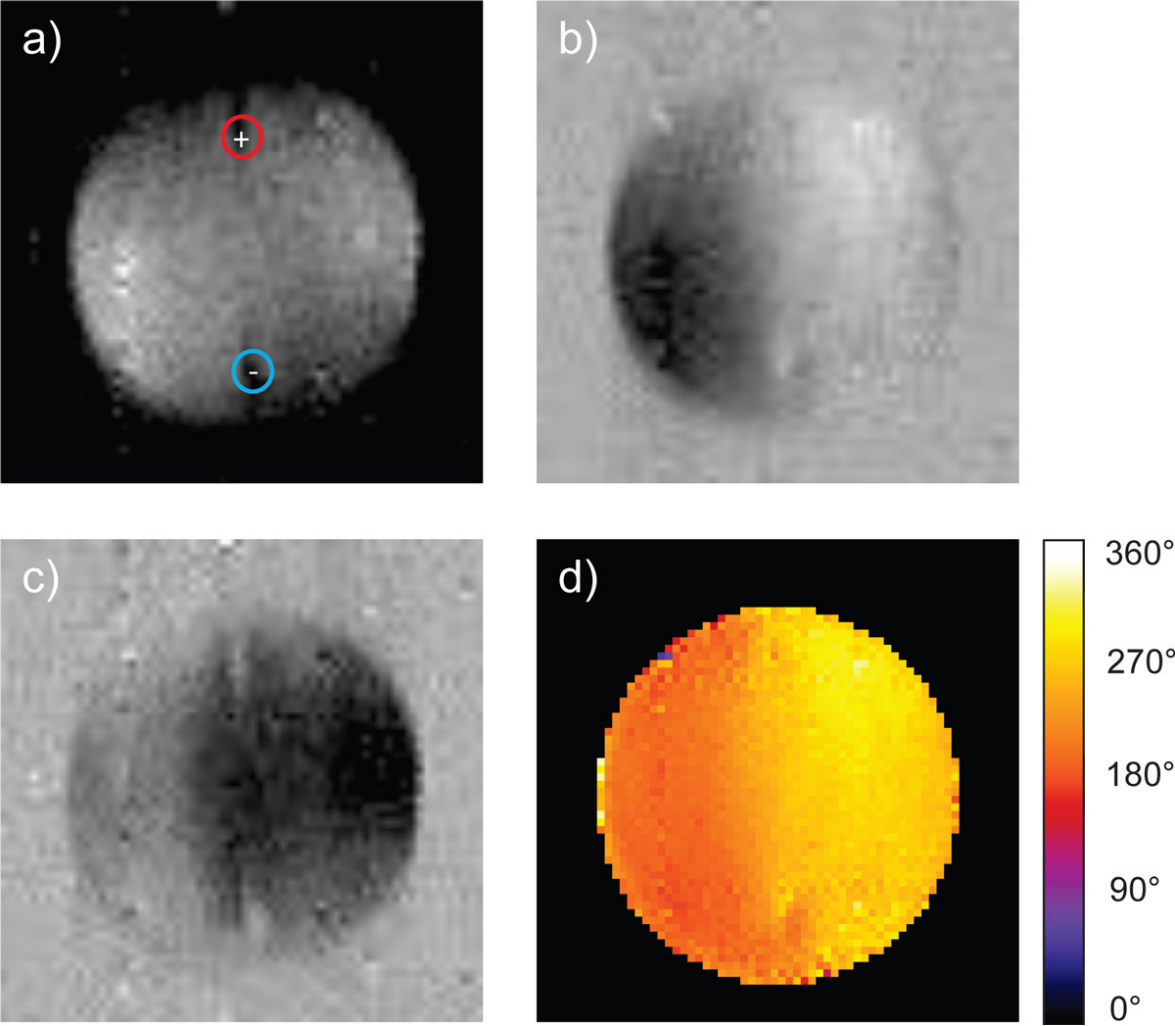
**Magnitude (a), real signal component (b), imaginary signal component (c), and (d) phase image of the liver tissue containing the disc-shaped test sample**. During the 3000 V and 100 μs long electroporation pulse, current was flowing vertically from the top to the bottom electrode. Positions of the electrodes are marked with red (positive electrode) and blue (negative electrode) circles in the magnitude image. Colorbar legend corresponds to the current induced phase.

**Figure 5:**
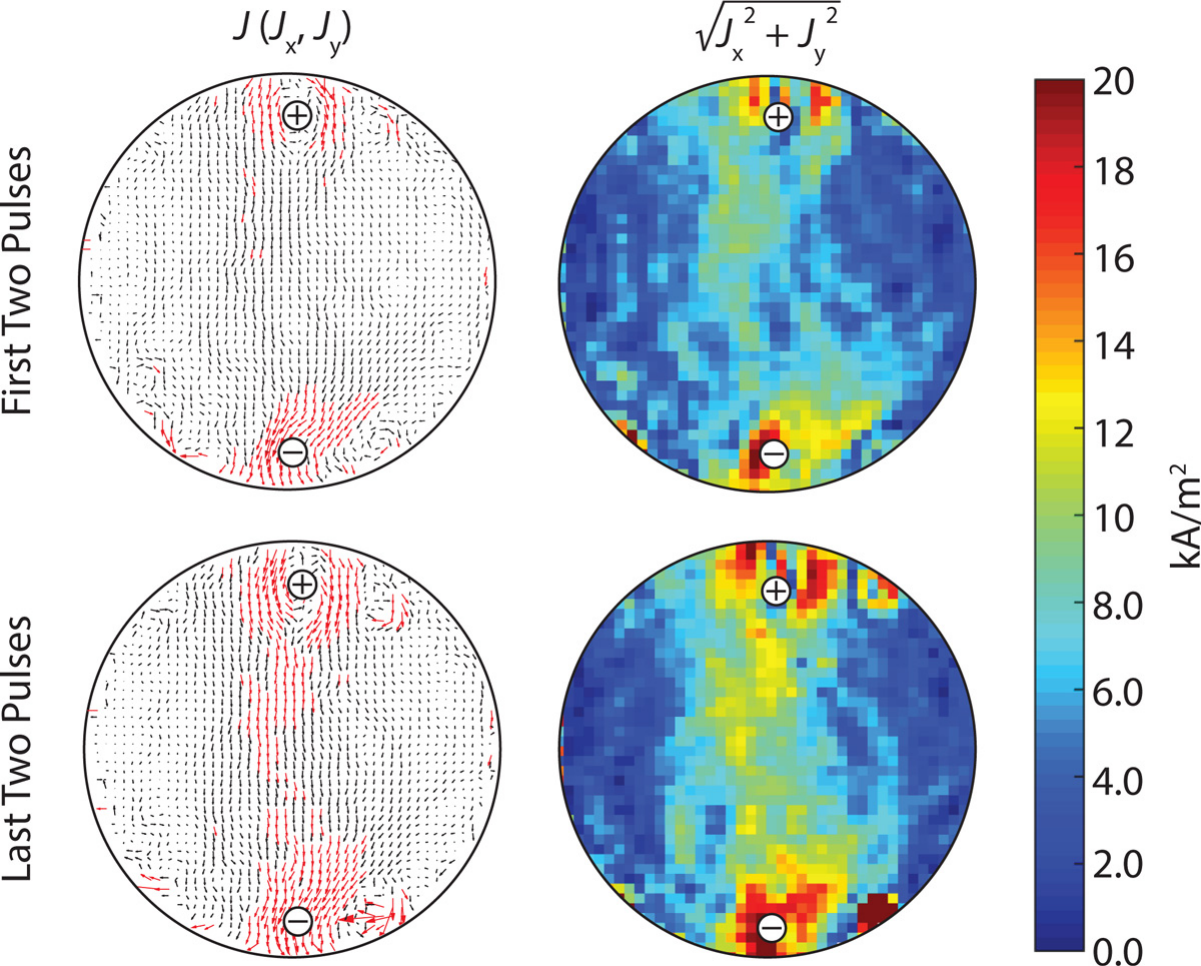
**Current density distribution obtained by CDI**. Tissues were exposed to 90 high voltage electric pulses with an amplitude of 3000 V and a duration of 100 μs at a repetition rate of 1 Hz. Pulses were delivered between two needle electrodes (marked with + and -). Current density distributions are presented as a vector field (two figures on the left; black and red arrows mark current density distributions that are lower and higher than 10 kA/m^2^, respectively) and as an absolute value (two figures on the right). Only current densities obtained during first two (pulse number 1 and 2) and last two pulses (pulse number 89 and 90) are presented for easier comparison.

When the tissue was exposed to a sequence of 90 electroporation pulses, i.e. a standard IRE sequence, *ex vivo *an electric current density was established inside the tissue. After each pair of successive electric pulses an electric current density distribution was successfully acquired by means of CDI two-shot RARE pulse sequence. Figure 4 presents magnitude, real and imaginary signal components as well as a phase image of the test sample during the electroporation pulse (3000 V, 100 μs, pulse number 89 and 90) acquired by the sequence. In the phase image Figure 4D it can be observed that the electric pulses induce a phase shift in the range 180-300°, which was more than the phase noise of 6.3° (measured as standard deviation of phase in the sample region of the phase image). In Figure 5 current density distribution during the first two (pulse number 1 and 2) and the last two pulses (pulse number 89 and 90) is presented. As it can be seen on vector field figures an area with higher current density (larger than 10 kA/m^2^) was established around the electrodes during the application of first two pulses whereas during the last two pulses this area has expanded towards the area between the electrodes. The direction of current path did not change with repeated electric pulses significantly, it only got more focused to the direct path between the electrodes. Thus, the current density in 10 × 10 pixels large area in the center of the tissue increased from the first to last electric pulses by 30%, i.e. from 6.6±0.3 kA/m^2 ^to 8.6±0.4 kA/m^2^.

**Figure 6:**
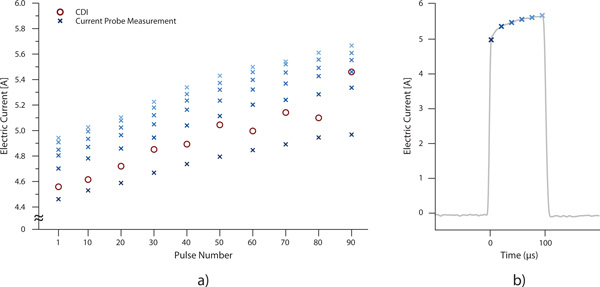
**Measured electric current during each of the applied electric pulses by means of current probe (marked with blue crosses) and reconstructed electric current by means of CDI (marked with red circles) during the application of IRE pulse sequence**. The CDI values were obtained as surface integrals of current density of over the area encircling one of the electrodes. Only the first and every tenth pulse are shown for easier comparison (a). The increase of the amplitude during each pulse is presented with 6 measurements obtained by the current probe. Here, measurements obtained on the last (90th) pulse are shown (b).

A comparison of measured electric current by means of the current probe and reconstructed electric current by means of CDI for one of the examples is shown on Figure 6. Average value of measured electric current by means of the current probe has increased from 4.8 A to 5.4 A from the first to the last pulse, respectively.

While, as shown in Figure 1, CDI has its peak sensitivity at *t_c _*= *T*_2_, unfortunately, in practice most biomedical applications of CDI are such that there is a demand for either application of very short and high current pulses or very long and low current pulses. The first case is associated with CDI applications in monitoring electroporation pulses which are considered in more detail in this work, while the second case is associated with attempts to image physiological currents which are low due to low conductivity of biological tissues and also due to low voltages that living systems can bear if applied externally or possess inherently [16]. The most challenging of these is undoubtedly CDI of neuronal currents, which is still mostly at the level of theoretical concepts [31,32]. The CDI sensitivity may become problematic in IRE experiments, where the standard IRE protocol uses 100 μs pulses repeated 90 times at the frequency of 1 Hz. The frequency of 1 Hz is too low to enable execution of more than one electric pulse in one CDI sequence. This limit is posed by the *T*_2 _relaxation of biological tissues, which is of the order of 100 ms. Only one 100 μs pulse is four times shorter than what was used in our previous experiments on the same test sample where four 100 μs were delivered in intervals of 100 μs, i.e. 5 kHz pulse repetition frequency [11]. Our previous experiments thus had four times better sensitivity. The only way to compensate the sensitivity loss due to shortening the electric pulses is to increase *SNR *of the image, which can be done by increasing imaging slice thickness or decreasing images resolution. Another option would be to perform experiments in a higher field magnet. Namely, image signal in theory increases approximately proportionally with the magnetic field squared. Alternatively, delivery of IRE sequence of pulses in another schedule could be considered, e.g. 4 or up to 10 pulses at high pulse repetition frequency.

Results presented in Figure 5 and 6 confirm that developed CDI sequence was successfully applied for measuring current density distribution during application of IRE pulse sequence. In Figure 5 two current densities distribution are compared; distribution established during first two pulses and during the last two. We can observe that current density increased at the end of the sequence compared to the density established in the beginning of the sequence. The increase of current density is expected since the tissue was exposed to the sequence of electric pulses that resulted in increase of electrical conductivity due to local tissue electroporation, which we believe is predominant effect but can also be attributed to the rise of temperature in the tissue (Joule heating) [13,33]. Also, in each of 45 obtained current density distributions a region between the electrodes where current density was higher compared to the rest of the tissue was observed. This was also the region where the electric field was the highest and electroporation process and Joule heating the strongest. After the experiment we observed damaged tissue between and around the electrodes by the naked eye. However, for more precise assessment of the tissue damage histological analysis of tissue samples should be performed.

Electric current was also measured by means of a current probe. In Figure 6A the increase of current during application of 90 pulses can be observed. As it can be observed in Figure 6A similar trend of current was also acquired by calculation of an absolute value of electric current density. It should be noted that the highest obtained value of electric current in this study was around 5.5 A while in clinical cases of *in vivo *IRE the current can reach up to 50 A [26] due to electrode depth of insertion. Application of CDI during *in vivo *IRE would therefore enable measurement of current density distributions with even better *SNR *than one presented in this study. In Figure 6B, it can also be observed that the current profile during the electroporation pulse does not have a spike at the beginning of the pulse [34]. This could be due to the use of a low-pass filter between the electric pulse generator and the electrodes in the sample. The filter, which is installed in the Faraday cage around the magnet, blocks all RF disturbances and other high-frequency signals which could spoil the NMR signal. In addition, acquisition bandwidth settings of the digital oscilloscope by which the current profile was recorded were inadequate for detection of the spikes.

CDI is the most important component of monitoring the electroporation process by means of MREIT since it provides current density distribution to MREIT algorithms for reconstruction of electric field distribution. Until now, CDI sequences could only be applied for acquiring current density distributions during delivery of electric pulses with repetition rate of 5 kHz and the whole acquisition took around 20 sec. New CDI sequence presented in this study is able to monitor current density distribution during the delivery of electric pulses with the repetition rate of 1 Hz and the acquisition time is shortened to a few seconds, thus new sequence can provide current density distribution every two pulses which provides opportunity for observation of current density distribution and electric field distribution during the IRE procedure. New CDI sequence was tested on a tissue sample *ex vivo*, however, its application *in vivo *seems feasible and will be our next research step. It is expected that such application would require only minor modifications of the experimental setup. Most of them will be associated with more difficult positioning of the electrodes in terms of their alignment along the direction of the static field and their interdistance. Namely, in this study one of few ideal electrode arrangement was used that do not require sample reorientation in the magnet to extract 2D current distribution. In the arrangement, the exposed parts of the electrodes were positioned in a plane perpendicular to the *B*_0 _field, while the electrodes were parallel to the *B*_0 _field. For the arrangement, the only nonzero current induced magnetic field component in the plane has *B*_0 _direction (it is perpendicular to the plane) and is therefore easy to detect. These conditions are often difficult to meet, especially in *in vivo *experiments where positioning of the electrodes is more difficult and the sample has a complex geometry. The sample reorientation in the magnet is a major limitation of CDI experiments, especially when performed *in vivo*, and is usually avoided by using electrode arrangements that do not require sample reorientation (along with the electrodes) in the magnet.

Monitoring of electroporation process through reconstruction of electric field distribution by means of MREIT was until now limited to only electroporation applications with an electric pulse repetition rate in the range of kHz, such as ECT, due to the frequency limitation of CDI sequences. The new CDI sequence widens the range of electroporation applications, including those with electric pulse repetition rate of around 1 Hz, as for example IRE tissue ablation. Determining current density after each pair of pulses would not only provide electric field determination but also enable follow-up of electric field distribution and electrical conductivity during the delivery of clinically relevant IRE sequence providing potentially important information that would allow interventional radiologist to monitor electroporation during intervention and perform corrective intervention if necessary.

## Conclusion

The presented single-shot RARE-based CDI sequence shows that current distribution imaging during delivery of short high-voltage electric pulses is feasible. As the sequence requires only two short electric pulses repeated in an interval of approximately 1 s, sequential imaging of current distribution during delivery of the IRE electric pulse train is possible. The method could potentially enable monitoring of tumor coverage by electric field during IRE tissue ablation.

## List of abbreviations

AC - alternating current

CDI - current density imaging

ECT - elctrochemotherapy

IRE - irreversible electroporation

MREIT - magnetic resonance electrical impedance tomography

MRI - magnetic resonance imaging

RARE - rapid acquisition with relaxation enhancement

RF - radiofrequency

## Competing interests

The authors declare that they have no competing interests.

## Authors' contributions

IS designed and programmed the modified CDI sequence that was used in this study and drafted the manuscript, MK prepared the tissue sample, calculate CDI images and drafted the manuscript, DM conceived and coordinated the study. All authors read and approved the final manuscript.

## Declarations

Publication costs for this article were sourced from Slovenian Research Agency (ARRS).

This article has been published as part of *BioMedical Engineering OnLine *Volume 14 Supplement 3, 2015: Select articles from the 6th European Conference of the International Federation for Medical and Biological Engineering (MBEC 2014). The full contents of the supplement are available online at http://www.biomedical-engineering-online.com/supplements/14/S3.
